# Cross-Cultural Adaptation and Validation of the Spanish Version of the Behavioral Regulation in Exercise Questionnaire for Children (BREQ-3C): Analysis of Psychometric Properties

**DOI:** 10.3390/healthcare13172197

**Published:** 2025-09-02

**Authors:** Raquel Pastor-Cisneros, Jorge Carlos-Vivas, José Francisco López-Gil, María Mendoza-Muñoz

**Affiliations:** 1 Physical Activity for Education, Performance and Health (PAEPH) Research Group, Faculty of Sport Sciences, University of Extremadura, 10003 Cáceres, Spain; raquelpc@unex.es (R.P.-C.); jorgecv@unex.es (J.C.-V.); 2 School of Medicine, Universidad Espíritu Santo, Samborondón 092301, Ecuador; 3 Vicerrectoría de Investigación y Postgrado, Universidad de Los Lagos, Osorno 5290000, Chile; 4 Department of Communication and Education, Universidad Loyola Andalucía, 41014 Sevilla, Spain; mamendozam@unex.es

**Keywords:** assessment, motivation, physical activity, self-determination theory, SPLA-C

## Abstract

Background/Objectives: In Spain, a high proportion of children do not meet the recommended daily levels of physical activity (PA), which highlights the urgent need to understand the motivational factors that could influence PA behavior. Self-Determination Theory is a widely used approach for assessing motivation toward exercise, employing instruments such as the Behavioral Regulation in Exercise Questionnaire (BREQ-3). However, despite the cognitive and linguistic differences that limit its direct application, this tool has not yet been adapted for children aged 6–12 years. This study aimed to adapt the BREQ-3 for use with Spanish schoolchildren and to evaluate its validity and reliability in this age group. Methods: The BREQ-3 for children (BREQ-3C) was linguistically and culturally adapted. Comprehension was tested through cognitive interviews, and reliability was assessed via a test–retest with 125 Spanish schoolchildren. Statistical analyses: Confirmatory factor analysis (CFA), Cronbach’s alpha, and the intraclass correlation coefficient (ICC) were used to evaluate validity and reliability. Results: CFA supported the factorial structure of the adapted BREQ-3 for primary schoolchildren, showing acceptable model fit indices (chi-square minimum discrepancy/degrees of freedom (CMIN/df) = 1.552, root mean square error of approximation (RMSEA) = 0.053, comparative fit index (CFI) = 0.891, Tucker-Lewis index (TLI) = 0.870). Internal consistency ranged from poor to excellent for all items and the total score of the questionnaire (Cronbach’s alpha (*α*): 0.535 to 0.911), except for items 3, 13, 20, and 21, where the internal consistency was unacceptable. Test–retest reliability was generally satisfactory, with ICC values indicating fair to excellent temporal stability (ICC: 0.248 to 0.911). The measurement error indicators (standard error of measurement percentage (SEM%) and minimal detectable change percentage (MDC%)) varied widely, particularly for the less reliable items. Most item scores were not significantly different between the test and retest groups, although items 2, 3, 5, 9, 17, 19, and 20 were significantly different. Conclusions: The BREQ-3C has promising psychometric properties for assessing exercise motivation in children aged 6–12 years. This tool shows potential for use in research, education, and health interventions to understand and promote physical activity motivation in primary schools.

## 1. Introduction

In Spain, the Physical Activity, Sedentary Behaviour, and Obesity in Spanish Youth (PASOS) study revealed that only 36.7% of children and adolescents comply with the World Health Organization’s recommendation of at least 60 min of moderate or vigorous physical activity (PA) per day [1]. As the promotion of PA has become a public health priority in our country [2], numerous researchers are seeking to understand the factors that influence and predict PA behavior, which is a topic of great interest to the scientific community [3,4,5]. For this reason, motivational factors have been extensively studied [6] via Self-Determination Theory as a theoretical reference [7].

Self-Determination Theory is a psychological theory that explains human motivation, personality development, and well-being [8], and it has also been applied to the study of exercise motivation [9,10]. Based on this theory, instruments have been developed to assess the relationship between motivation and different types of PA, such as exercise or sport. Examples include the Perceived Locus of Causation Questionnaire [11], the Behavioral Regulation in Sport Questionnaire [12], and the Behavioral Regulation in Exercise Questionnaire (BREQ) [13]. These instruments have been updated and translated into several languages to expand their use.

The BREQ was originally created to assess levels of motivation toward physical exercise on the basis of Self-Determination Theory [13], resulting in more complete versions, such as the BREQ-2 [14] and the most recent version, the BREQ-3, which includes 23 items grouped into six dimensions covering the continuum of Self-Determination Theory from amotivation to intrinsic motivation and has adequate fit indicators [15]. Furthermore, evidence of its application, adequacy, and consistency has been supported in adult populations [16] and young university students [17].

Specifically, the BREQ-3 was originally applied to a general population of adults and adolescents who practice PA [18,19,20], but other studies have also validated its use in different contexts for specific populations (e.g., older adults) [21,22], showing that it can be adapted for use with different groups. In addition, it has also been used with children aged 10–12 years [23,24]. However, given that our target population is children aged 6–12, and to our knowledge, no study has included an adaptation for this age group; there is currently a gap in the literature.

In this sense, the original instrument needs to be adapted instead of being used directly, given the sociocultural and contextual differences [25,26] as well as the cognitive and linguistic differences [27] found between the adolescent–adult population and school-aged children. This will allow us to more accurately assess the motivations of children in this population for PA.

Given the significant developmental differences between school-aged children and older populations [28], it is crucial to develop a version of the BREQ-3 that is more accessible and understandable for children by adjusting the language, structure, and items. This study, therefore, aims to adapt the BREQ-3 instrument for use with Spanish schoolchildren aged 6–12 years and to assess the validity and reliability of the adapted instrument.

## 2. Materials and Methods

### 2.1. Study Design

This instrumental, methodological study aimed to linguistically and culturally adapt the BREQ-3 for use with children aged 6–12 years. The original version of the questionnaire was designed for adolescents and adults (Behavioral Regulation in Exercise Questionnaire for Children—BREQ-3C). The reason for selecting this age range was to encompass the primary education stage. This study also aimed to analyze the psychometric properties of the questionnaire (validity and reliability) in this new population.

This study was carried out in three main phases:

(1) Linguistic and cultural adaptation: The vocabulary, sentence structure, and content of the original instrument were adapted to suit the cognitive, linguistic, and socio-emotional developmental levels of school-aged children. This process involved adapting and reformulating each item, which was then reviewed by three experts in children’s physical literacy (PL), education, and motivation in the field of PA.
(2) Assessment of comprehension through cognitive interviews: To ensure the clarity and appropriateness of the items, individual cognitive interviews were conducted with a pilot sample of children. The literal and contextual comprehension of the items was analyzed during these interviews.
(3) Test–retest reliability study: A subsample of participants completed the questionnaire twice, 2 weeks apart, to assess temporal response stability by analyzing test–retest reliability.

This design enabled rigorous adaptation of the instrument to children’s developmental characteristics to assess its content validity, functional comprehension, and temporal consistency. The adapted instrument, BREQ-3C, can be found in Supplementary Materials S1.

### 2.2. Participants

A total of 125 schoolchildren (61 boys and 64 girls) aged between 6 and 12 years were recruited from various primary schools in Extremadura, Spain. Participants were selected via non-probability convenience sampling. Inclusion criteria included being enrolled in primary education, having provided informed consent signed by a parent or legal guardian, and having an adequate level of reading comprehension to complete the adapted questionnaire.

The target age range of 6–12 years was selected to encompass the full span of primary education in Spain, corresponding to the intended scope of the BREQ-3C within the SPLA-C model. The instrument was designed to be comprehensible and relevant to all children in this stage, regardless of grade or developmental phase. Cognitive interviews confirmed that items were understandable across the entire range. Children in first grade were excluded because, when assessed early in the academic year, they have not yet completed the physical education (PE) curriculum, potentially limiting their understanding of sport-related situations. The aim was therefore to create a unified tool applicable to the whole primary school population with prior exposure to PE, enabling consistent assessment in both research and pedagogical contexts.

### 2.3. Ethics Approval

This study involved human participants and was approved by the Bioethics and Biosafety Committee of the University of Extremadura (approval number 288/2024). The updates of the Declaration of Helsinki, amended by the 75th General Assembly of the World Medical Association (Helsinki, Finlandia, 2024) and Law 14/2007 on Biomedical Research, were followed. The participants provided informed consent to participate in this study before taking part.

### 2.4. Instrument and Adaptation Process

The research team collected data using the adapted version of the BREQ-3C, which is designed to assess different types of motivational regulation in the context of PA based on Self-Determination Theory.

The Spanish version of the BREQ-3 [15] was used to collect data on the different types of motivational regulation in the context of PA, and it comprises 23 items distributed across six subscales on the basis of Self-Determination Theory: intrinsic motivation, identified regulation, integrated regulation, introjected regulation, external regulation, and demotivation.

The BREQ-3 score is obtained by calculating the average of the items that make up each of the six subscales: amotivation (items 6, 11, 14, 23), external regulation (items 1, 7, 13, 19), introjected regulation (items 2, 8, 16, 21), identified regulation (items 3, 9, 17), integrated regulation (items 5, 10, 15, 20), and intrinsic motivation (items 4, 12, 18, 22). Each item is answered on a 5-point Likert scale, from 0 (‘not at all true for me’) to 4 (‘completely true for me’), so the scores for each subscale range from 0 to 4. The Relative Autonomy Index (RAI) can also be calculated by weighting the means of each subscale with the following coefficients: intrinsic motivation × +3, integrated regulation × +2, identified regulation × +1, introjected regulation × −1, external regulation × −2, and amotivation × −3. The resulting RAI reflects the degree of self-determination of motivation, with higher values indicating greater self-determined motivation.

To adapt the instrument for use with children, the BREQ-3 items underwent a process of linguistic and cultural adaptation (see Table 1). This involved (a) simplifying the vocabulary and syntax and (b) eliminating or replacing abstract or ambiguous concepts.

The BREQ-3 was adapted to create the BREQ-3C, which retains the original theoretical dimensions. As the adaptation was based on an established structure, a confirmatory factor analysis (CFA) was conducted to determine whether the original factor structure remained valid for the new population. A panel of experts reviewed the content of each item, and cognitive interviews were conducted with a pilot group of children (n = 17) to check the clarity, comprehension, and appropriateness of each item. Notably, the wording of the items remained unchanged after the cognitive interviews.

### 2.5. Statistical Analyses

All the data gathered were recorded in a database designed for this research project, and personal data were kept anonymous. Statistical analyses were performed via the Statistical Package for the Social Sciences (SPSS, version 25.0; IBM SPSS Inc., Armonk, NY, USA). The software package AMOS v.23.0.0 (IBM Corporation, Wexford, PA, USA) was used to perform CFA. The different items of the BREQ-3C (Spanish version) were included as elements. To assess the model’s goodness of fit, the following indices were selected: (1) the chi-square probability setting as appropriate nonsignificant values (*p* > 0.05) [29], (2) the root mean square error of approximation (RMSEA) [30], (3) the comparative fit index (CFI), (4) the Tucker-Lewis Index (TLI), and (5) the chi-square per degree of freedom ratio (chi-square minimum discrepancy/degrees of freedom (CMIN/df)) [31].

A test–retest reliability check was then performed 15 days later. The data are presented as the means and standard deviations (SDs) for the initial and follow-up assessments. The Shapiro-Wilk and Levene tests were used to verify normality and equality of variance for all the variables that were assessed. The internal consistency and reliability of each item in the BREQ-3C and the overall score were evaluated via Cronbach’s alpha (α) coefficient. According to Glen (2022) [32], the interpretation of Cronbach’s *α* is as follows: <0.5 signifies unacceptable; 0.5 to 0.6 indicates poor; 0.6 to 0.7 suggests questionable; 0.7 to 0.8 is acceptable; 0.8 to 0.9 is good; and >0.9 represents excellent. Test–retest reliability or consistency was evaluated by computing the intraclass correlation coefficient (ICC) with a 95% confidence interval [33]. A two-way random effects model, single measures, absolute agreement, and the ICC were used to demonstrate agreement between the two tests. ICC scores were interpreted based on the standards provided by Landis and Koch [34]: <0.20 indicates slight agreement, 0.20 to 0.39 indicates fair agreement, 0.40 to 0.60 indicates moderate agreement, 0.61 to 0.80 indicates substantial agreement, and >0.80 indicates near perfection. Additionally, the standard error of measurement (SEM) and minimum detectable change (MDC) [35] were used to assess absolute reliability. Finally, because the data did not follow a normal distribution, Spearman’s rho (*ρ*) was used to determine the relationship between each item and the total score. The significance level was set at *p* < 0.05 for all tests.

## 3. Results

### 3.1. Confirmatory Factor Analysis

**Figure 1:**
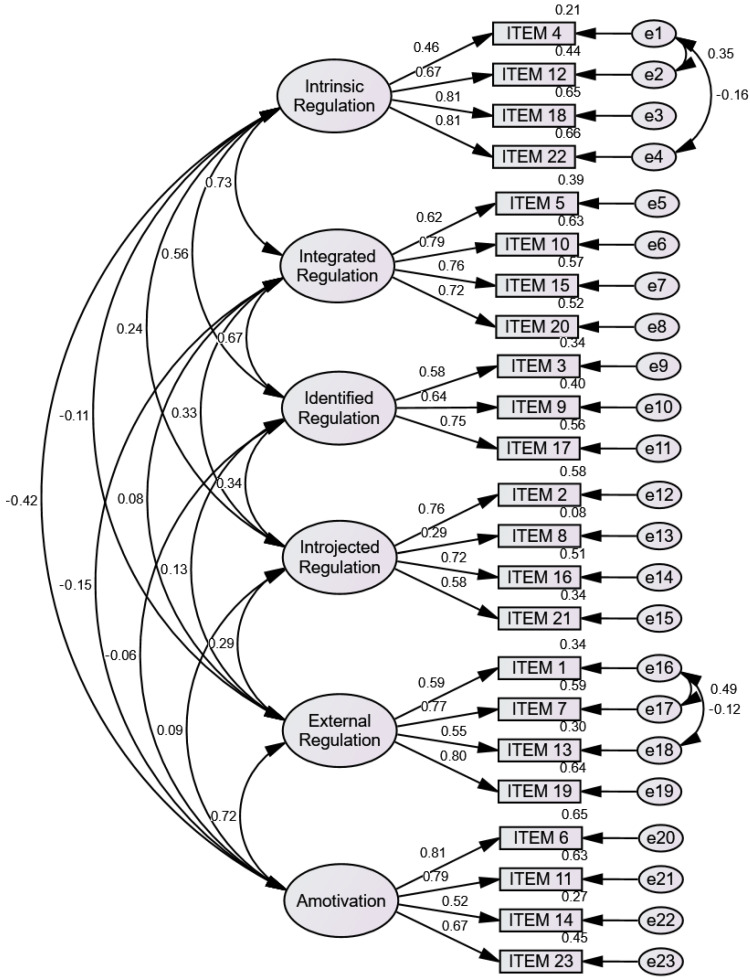
CFA resulting model for BREQ-3C.

A CFA was conducted with a total of 125 participants aged 9.63 (SD = 1.78) years, of whom 49.6% were girls. Figure 1 displays the resulting model from the CFA. To aid interpretation of the measurement model, we highlight that certain items exhibited lower factor loadings with their respective subscales. Specifically, item 4 (intrinsic regulation), item 3 (identified regulation), item 8 and item 21 (introjected regulation), item 1 and item 19 (external regulation), and item 14 (amotivation) all showed factor loadings below 0.50. By explicitly linking these questions to their associated subscales, we aim to facilitate the reader’s understanding of which items may be less representative within the overall scale structure (see Figure 1).

The goodness-of-fit indices following CFA are shown in Table 2. Overall, the model fit was deemed acceptable by the CFA. A good fit in relation to model complexity was indicated by the CMIN/df of 1.552 [36]. The TLI was 0.870, and the CFI was 0.891, both of which were within a marginally acceptable range but slightly below the traditional cutoff of 0.90 [37]. Given that values below 0.08 are typically regarded as acceptable, the RMSEA of 0.067 indicated a reasonable error of approximation [38]. Although small model adjustments could further enhance the fit, these results confirm that the suggested factorial structure is adequate.

### 3.2. Test–Retest Reliability and Internal Consistency

Table 3 shows the internal consistency, reproducibility, and systematic differences of the BREQ-3C. Overall, the internal consistency ranged from poor to excellent for all items and the total score of the questionnaire (Cronbach’s α from 0.535 to 0.911), except for items 3, 13, 20, and 21, where the internal consistency was unacceptable (Cronbach’s α from 0.404 to 0.497). All the items in the initial and follow-up tests were significantly correlated with the total BREQ-3C score (Spearman’s *ρ* > 0.3), except for items 2, 16, and 21.

The reproducibility outcomes revealed fair-to-near perfection test–retest reliability for each item and the total BREQ-3C score (ICC: 0.248 to 0.911). The SEM and SEM% values for each item and the total BREQ-3C score ranged from 0.65 to 1.89 and from 12.02 to 223.02, respectively. Similarly, the MDC and MDC% values for each item and the total BREQ-3C score ranged from 1.79 to 5.25 and from 33.32 to 618.18, respectively.

Finally, the comparison between test and retest scores showed no significant differences for any of the items or the total BREQ-3C score (*p* > 0.05), except for item 2 (*p* = 0.043), item 3 (*p* = 0.035), item 5 (*p* = 0.002), item 9 (*p* = 0.041), item 17 (*p* = 0.043), item 19 (*p* = 0.013), and item 20 (*p* = 0.050).

## 4. Discussion

The primary aim of this study was to adapt and validate the BREQ-3C for use with Spanish-speaking schoolchildren aged 6–12, taking into account the significant developmental differences between this age group and older populations. The results of the cross-cultural adaptation and psychometric evaluation suggest that the adapted version is valid and reliable for this age group. The results of the CFA supported the proposed factorial structure, with fit indices falling within acceptable or marginally acceptable ranges. In terms of reliability, the adapted questionnaire demonstrated fair to excellent test–retest reliability, with adequate internal consistency for most items and the overall scale. However, a few items exhibited lower internal consistency, suggesting the need for further refinement. Despite these limitations, no significant systematic differences were observed over time for most items, supporting the instrument’s temporal stability. Overall, these findings suggest that the adapted BREQ-3C is a promising instrument for assessing motivational regulation towards exercise in primary schoolchildren, with valuable applications in research, education, and healthcare.

The CFA results support the proposed factorial structure of the adapted BREQ-3C for primary schoolchildren. The chi-square to degrees of freedom ratio indicated a good fit relative to model complexity, aligning with commonly accepted standards. While the TLI (0.870) and CFI (0.891) were marginally below the conventional threshold of 0.90, they remained within the generally accepted range. Similarly, the RMSEA (0.067) indicated reasonable approximation error, remaining below the upper cutoff of 0.08. Overall, these results suggest that the model adequately represents the data structure among children aged 6–12, particularly given the challenges associated with adapting psychological instruments for younger populations. Previous validation studies of the BREQ-3 in adolescent and adult samples have reported similar difficulties in achieving optimal fit indices, particularly in cross-cultural contexts [18,39]. Therefore, the slight deviations observed in this study are not uncommon and are likely attributable to developmental and linguistic differences inherent in working with a younger sample.

Furthermore, while minor model refinements could improve fit indices, the current structure appears to capture the key motivational dimensions outlined in self-determination theory [7]. This finding supports the theoretical soundness and practical applicability of the adapted version in assessing exercise motivation among schoolchildren.

The test–retest reliability of the adapted BREQ-3C was generally satisfactory, with ICC values ranging from 0.248 to 0.911. These results suggest that most items demonstrated fair to excellent temporal stability, which is consistent with previous validation studies of the BREQ-3 in adolescent and adult populations [15,40,41], where ICCs typically fall within similar ranges. However, the lower ICCs observed for certain items may be influenced by the age-related variability in cognitive and emotional development in younger children [42], who still consolidate their understanding of internal motivational states. These results suggest that these items may be less consistent in capturing the intended construct over time, potentially due to age-related comprehension challenges or the abstract nature of the statements. Despite this, we retained them in the present version because they address theoretically relevant aspects of motivation and contribute to the conceptual integrity of their respective subscales. Removing them could risk narrowing the construct coverage of the BREQ-3C. Instead, we recommend that future research examine these items in larger and more diverse samples and explore whether rewording or simplifying the language could enhance their reliability without compromising content validity.

Indicators of measurement error, such as SEM% and MDC%, varied widely (SEM%: 12.02 to 223.02; MDC%: 33.32 to 618.18), particularly for items with lower reliability (e.g., items 6, 13, 14, 19, 21, and 23). These high values may reflect the difficulty of measuring stable motivational constructs in a young population whose abstract thinking and self-awareness are still developing. Similar findings have been reported in the adaptation of motivational instruments for children elsewhere, where the precision of change detection tends to be lower than in adult samples [43].

Nevertheless, the total BREQ-3C score demonstrated consistent reliability over time, indicating that, although some items may need refining, the instrument as a whole is sufficiently stable for use in research and educational contexts involving children. Continued adaptation efforts focusing on clarity of wording and developmental appropriateness could help to reduce measurement error in future versions.

The comparison of item scores between the test and retest revealed no significant differences for most items, reinforcing the temporal stability of the adapted questionnaire.

However, statistically significant differences were found for items 2, 3, 5, 9, 17, 19, and 20. Importantly, the majority of these items belong to the identified regulation subscale, which overall showed lower reliability compared with the other subscales. This finding indicates that the identified regulation dimension was more prone to measurement instability and therefore performed worse than the others. A likely explanation is that items assessing identified regulation often involve more abstract, internalized reasons for exercising (e.g., acting according to personal values), which may be difficult for children in this age range to articulate or fully comprehend [44]. This interpretation is consistent with previous research [45], which has shown that younger children struggle with the cognitive demands of self-report measures derived from self-determination theory, particularly when items are not tailored to their developmental stage.

Finally, the presence of small but significant differences in repeated measures could reflect environmental influences [46], such as changes in school routines or mood, which tend to impact younger participants more strongly. Therefore, variability in these items may not necessarily indicate flaws in the instrument, but rather natural fluctuations in children’s responses.

### 4.1. Strengths and Practical Implications

The findings of this study have several important implications for researchers and practitioners working with children in educational, sporting, or health-related settings. First, the successful adaptation of the BREQ-3C provides a developmentally appropriate means of assessing motivational regulation in PA contexts among Spanish-speaking primary schoolchildren. This enables more nuanced assessments of motivation based on Self-Determination Theory, which is central to designing interventions that foster autonomous motivation and long-term adherence to PA [8,43].

In practice, this tool can help physical educators and psychologists to identify students who are at risk of having low or externally regulated motivation and to adapt programs accordingly. From a research perspective, having a valid and reliable measure available allows for more robust data collection in longitudinal, intervention, and cross-cultural studies involving younger populations.

Importantly, this study aligns with a recent article on the development of the first assessment model for PL in Spain (i.e., the SPLA-C) [46]. In this Delphi study, national experts decided that this questionnaire should be part of the ‘motivation and confidence’ component, forming part of this new assessment model for PLs in Spain. As part of the SPLA-C model, the BREQ-3C allows schools and policy makers to monitor students’ development in the affective domain of PL.

The BREQ-3C can be readily integrated into school-based PE programs and national monitoring systems, such as the SPLA-C, to identify students’ motivational profiles and inform targeted interventions. For example, a PE teacher could administer the BREQ-3C at the beginning of the school year to identify students with low autonomous motivation toward PA. Based on these results, the teacher might implement strategies such as offering greater choice in activities, emphasizing personal progress, and creating more supportive peer environments. At the policy level, aggregated BREQ-3C data could be incorporated into national fitness or PL assessments to monitor motivational trends across regions and age groups, enabling the development of tailored initiatives to foster lifelong PA engagement. By linking motivational assessment with instructional and policy decisions, the BREQ-3C serves as a valuable tool for both educational practice and large-scale monitoring.

### 4.2. Limitations and Future Line Research

Despite the promising results, several limitations must be acknowledged. First, the sample was limited to a specific geographical region of Spain, which may restrict generalizability to broader Spanish-speaking populations. Therefore, future studies should replicate the validation process using more diverse and representative samples.

Second, the nature of this study precludes conclusions about predictive validity or sensitivity to change over time. Future research should adopt longitudinal and intervention-based designs to determine the extent to which the instrument captures changes in motivation over time.

Third, although the CFA supported the proposed structure, certain fit indices were slightly below conventional thresholds. This suggests that item wording needs to be refined further to enhance developmental appropriateness. Additionally, consideration could be given to removing items that do not meaningfully contribute to their respective dimensions or the overall score. This would improve the psychometric properties of the instrument and result in a simpler, shorter, and more accessible tool for young children.

Furthermore, some psychometric indicators, particularly test–retest reliability and internal consistency of specific items, were suboptimal, likely due to developmental factors. It is possible that younger children have not yet developed the cognitive abilities needed to interpret motivational statements, as these are more complex and abstract. Future adaptations should therefore consider using simpler language, concrete examples, or visual aids to facilitate comprehension and reduce variability.

When using the current version of the BREQ-3C, researchers and practitioners should exercise caution when interpreting the results of items with lower reliability or temporal stability. At the individual level, single-item scores from these questions may be less precise, so reliance on subscale or total scores is recommended instead, since these aggregate measures have acceptable psychometric properties. At the group level, these items can still contribute meaningfully to the detection of trends, provided that potential measurement error is acknowledged in analyses and interpretations. Future research should test alternative formats or modified wording for these items to improve their psychometric strength. Additionally, as the present sample was drawn from a single Spanish region, replication in different cultural and geographical contexts is necessary before generalizing the results to all Spanish-speaking child populations. Until such data are available, findings should be interpreted in light of the characteristics of the study sample.

## 5. Conclusions

The Spanish adaptation of the BREQ-3C shows promising psychometric properties for evaluating exercise motivation in children aged 6–12. The instrument demonstrated an acceptable overall factorial structure, as supported by the CFA results. Test–retest reliability was generally fair to excellent, indicating good temporal stability. Internal consistency was adequate for most subscales and the total score. Although some items showed lower reliability and significant differences over time, this is likely due to the evolving cognitive and emotional capacities of schoolchildren. Measurement error varies across items, highlighting the need for further refinement, particularly for items with more abstract content. The BREQ-3C holds promise for application in research, education, and health-related interventions aimed at understanding and promoting PA motivation in primary school settings. Future research should refine item wording further and test the instrument in broader and more diverse child populations to increase its developmental sensitivity and psychometric robustness. The BREQ-3C forms part of the affective domain of the SPLA-C, the first model for the assessment of PL in Spain.

## Figures and Tables

**Table 1:** Adaptation process of the BREQ-3C.

Items	Spanish Version of BREQ-3 (Original)	Final Version (BREQ-3C) Agreed Version Through Internal Consensus
Opening sentence	*Yo hago ejercicio físico…*	*Yo hago ejercicio/deporte…*
1	*Porque los demás me dicen que debo hacerlo*	*Porque los demás me dicen que tengo que hacerlo*
2	*Porque me siento culpable cuando no lo practico*	*Porque me siento mal cuando no lo hago*
3	*Porque valoro los beneficios que tiene el ejercicio físico*	*Porque sé que es bueno para mí*
4	*Porque creo que el ejercicio es divertido*	*Porque creo que es divertido*
5	*Porque está de acuerdo con mi forma de vida*	*Porque va bien con mi forma de vivir/ser*
6	*No veo por qué tengo que hacerlo*	*No creo que tenga que hacer deporte o ejercicio*
7	*Porque mis amigos/familia/pareja me dicen que debo hacerlo*	*Porque mis amigos y mi familia me dicen que tengo que hacerlo.*
8	*Porque me siento avergonzado si falto a la sesión*	*Porque me da vergüenza si no voy a un entrenamiento/clase*
9	*Porque para mí es importante hacer ejercicio regularmente*	*Porque para mi es importante hacer ejercicio o deporte cada semana*
10	*Porque considero que el ejercicio físico forma parte de mí*	*Porque creo que el deporte o ejercicio es parte de mi*
11	*No veo por qué tengo que molestarme en hacer ejercicio*	*No entiendo por qué tengo que hacer ejercicio o deporte*
12	*Porque disfruto con las sesiones de ejercicio*	*Porque disfruto haciéndolo*
13	*Porque otras personas no estarán contentas conmigo si no hago ejercicio*	*Porque a los demás no les gustará si no hago ejercicio o deporte*
14	*No veo el sentido de hacer ejercicio*	*No veo por qué es importante hacer ejercicio*
15	*Porque veo el ejercicio físico como una parte fundamental de lo que soy*	*Porque veo que es una parte muy importante de mí*
16	*Porque siento que he fallado cuando no he realizado un rato de ejercicio*	*Porque me siento mal si no lo hago*
17	*Porque pienso que es importante hacer el esfuerzo de ejercitarse regularmente*	*Porque creo que es importante esforzarse para hacer ejercicio o deporte semanalmente*
18	*Porque encuentro el ejercicio una actividad agradable*	*Porque me gusta mucho hacerlo*
19	*Porque me siento bajo la presión de mis amigos/familia para realizar ejercicio*	*Porque mis amigos o familia me dicen que tengo que hacerlo/me obligan*
20	*Porque considero que el ejercicio físico está de acuerdo con mis valores*	*Porque creo que el ejercicio o deporte va con lo que pienso que es bueno*
21	*Porque me pongo nervioso si no hago ejercicio regularmente*	*Porque me pongo nervioso si no lo hago semanalmente*
22	*Porque me resulta placentero y satisfactorio el hacer ejercicio*	*Porque me hace sentir bien*
23	*Pienso que hacer ejercicio es una pérdida de tiempo*	*Pienso que hacer ejercicio deporte no vale la pena*

**Table 2:** BREQ-3C goodness-of-fit indices.

Indices	Value
CMIN/df	1.552
*p*-value (*χ*^2^)	<0.001
RMSEA	0.053
CFI	0.891
TLI	0.870

CMIN/df, minimum discrepancy per degree of freedom; *p*-value (χ^2^), chi-square probability; RMSEA, root mean square error of approximation; CFI, comparative fit index; TLI, Tucker-Lewis Index.

**Table 3:** Reliability, test-retest, and systematic differences in the BREQ-3C.

Item	Test (n = 125)			Retest (n = 125)			Reliability Test						
	M	SD	Item–Total Correlation	M	SD	Item–Total Correlation	Cronbach’s α	ICC (95% CI)	*p*-Value †	SEM	%SEM	MDC	MDC%
Item 1	0.70	1.24	−0.419 **	0.86	1.37	−0.530 **	0.544	0.373 (0.212 to 0.514)	0.245	1.03	132.5	2.86	367.2
Item 2	1.55	1.43	0.000	1.85	1.58	−0.091	0.598	0.420 (0.266 to 0.554)	0.043	1.15	67.4	3.18	186.9
Item 3	3.68	0.79	0.220 *	3.47	0.98	0.418 **	**0.404**	**0.248 (0.080 to 0.403)**	0.035	0.77	21.5	2.13	59.5
Item 4	3.14	1.22	0.486 **	3.18	1.11	0.541 **	0.711	0.553 (0.418 to 0.664)	0.684	0.78	24.6	2.16	68.3
Item 5	2.63	1.35	0.576 **	3.00	1.23	0.524 **	0.657	0.472 (0.319 to 0.600)	0.002	0.94	33.3	2.60	92.3
Item 6	0.35	0.90	−0.530 **	0.25	0.74	−0.312 **	0.553	0.381 (0.222 to 0.521)	0.205	0.65	215.0	1.79	596.1
Item 7	0.86	1.34	−0.486 **	0.89	1.27	−0.486 **	0.711	0.553 (0.418 to 0.664)	0.827	0.87	99.7	2.42	276.4
Item 8	0.51	1.23	−0.266 **	0.61	1.14	−0.340 **	0.725	0.568 (0.437 to 0.676)	0.307	0.78	139.1	2.16	385.5
Item 9	3.32	1.02	0.415 **	3.10	1.23	0.469 **	0.636	0.460 (0.311 to 0.587)	0.041	0.83	25.8	2.29	71.4
Item 10	2.98	1.22	0.552 **	3.03	1.24	0.585 **	0.755	0.608 (0.485 to 0.708)	0.622	0.77	25.6	2.13	71.0
Item 11	0.34	0.96	−0.507 **	0.41	0.99	−0.533 **	0.716	0.558 (0.425 to 0.668)	0.380	0.65	172.9	1.80	479.1
Item 12	3.46	1.06	0.576 **	3.46	0.97	0.537 **	0.733	0.580 (0.451 to 0.686)	>0.999	0.66	19.0	1.82	52.7
Item 13	0.52	1.15	−0.382 **	0.47	1.11	−0.433 **	**0.436**	**0.280 (0.109 to 0.434)**	0.692	0.96	193.7	2.66	536.9
Item 14	0.31	0.91	−0.396 **	0.42	1.11	−0.357 **	0.650	0.480 (0.334 to 0.604)	0.227	0.73	199.5	2.02	553.1
Item 15	3.15	1.18	0.469 **	3.18	1.22	0.464 **	0.663	0.498 (0.353 to 0.619)	0.766	0.85	26.9	2.36	74.5
Item 16	1.75	1.57	−0.082	1.84	1.65	−0.068	0.535	0.367 (0.204 to 0.509)	0.587	1.28	71.4	3.55	197.8
Item 17	3.24	1.05	0.459 **	3.02	1.19	0.456 **	0.577	0.399 (0.243 to 0.536)	0.043	0.87	27.7	2.41	76.9
Item 18	3.48	0.95	0.571 **	3.46	1.02	0.622 **	0.721	0.565 (0.432 to 0.674)	0.770	0.65	18.7	1.80	51.9
Item 19	0.70	1.31	−0.531 **	0.42	0.93	−0.369 **	0.541	0.361 (0.201 to 0.503)	0.013	0.90	159.9	2.48	443.2
Item 20	3.22	1.10	0.469 **	2.97	1.28	0.581 **	**0.474**	**0.306 (0.141 to 0.455)**	0.050	0.99	32.0	2.75	88.8
Item 21	0.93	1.23	−0.063	1.12	1.40	−0.171	**0.497**	**0.329 (0.165 to 0.476)**	0.160	1.08	105.1	2.99	291.3
Item 22	3.26	1.16	0.632 **	3.30	1.04	0.545 **	0.684	0.522 (0.382 to 0.639)	0.678	0.76	23.2	2.11	64.3
Item 23	0.30	0.95	−0.382 **	0.30	0.95	−0.357 **	0.668	0.504 (0.360 to 0.624)	>0.999	0.67	223.0	1.85	618.2
Total BREQ-3C score	15.86	6.22	N/A	15.63	6.47	N/A	0.911	0.911 (0.873 to 0.937)	0.494	1.89	12.0	5.25	33.3

Abbreviations: M, mean; SD, standard deviation; 95% CI, confidence interval of 95%; ICC, intraclass correlation coefficient; SEM, standard error of measurement; %SEM, standard error of measurement as a percentage; MDC, minimum detectable change; N/A, not applicable; †, Friedman test *p*-values. The item–total correlation refers to the magnitude of the association between each item and its domain. ** Significant correlation at *p* < 0.01. * Significant correlation at *p* ≤ 0.05. Values in **bold** indicate low reliability or internal consistency.

## Data Availability

Data available on request due to restrictions: The database will be available upon request to the corresponding author. The reason for the privacy of the data is because it involves minors.

## Supplementary Materials

The following supporting information can be downloaded at https://www.mdpi.com/article/10.3390/healthcare13172197/s1: Supplementary Materials S1: Behavioral regulation in exercise questionnaire for children (BREQ-3C)-Spanish and English (not validated) version.

## Abbreviations

The following abbreviations are used in this manuscript:

BREQ-3	Behavioral Regulation in Exercise Questionnaire
CFA	Confirmatory factor analysis
CFI	Comparative fit index
ICC	Intraclass correlation coefficient
SPLA-C	Spanish Physical Literacy Assessment for Children
PA	Physical activity
PE	Physical education
PL	Physical literacy
RMSEA	Root mean square error of approximation
TLI	Tucker-Lewis index

## References

[1] Gómez S., Lorenzo L., Ribes C., Homs C. (2019). Informe Estudio PASOS 2019.

[2] Rial-Vázquez J., Pérez-Ríos M., Varela-Lema L., Rey-Brandariz J., Candal-Pedreira C., Mourino N., Vila-Farinas A., López-Pardo E., Ruano-Ravina A. (2023). La actividad física en los planes de salud autonómicos de España. Una revisión de propuestas. Gac. Sanit..

[3] Hu D., Zhou S., Crowley-McHattan Z.J., Liu Z. (2021). Factors That Influence Participation in Physical Activity in School-Aged Children and Adolescents: A Systematic Review from the Social Ecological Model Perspective. Int. J. Environ. Res. Public Health.

[4] Hartman C.L., Barcelona R.J., Trauntvein N.E., Hall S.L. (2020). Well-Being and Leisure-Time Physical Activity Psychosocial Factors Predict Physical Activity among University Students. Leis. Stud..

[5] Martins J., Costa J., Sarmento H., Marques A., Farias C., Onofre M., Valeiro M.G. (2021). Adolescents’ Perspectives on the Barriers and Facilitators of Physical Activity: An Updated Systematic Review of Qualitative Studies. Int. J. Environ. Res. Public Health.

[6] Kalajas-Tilga H., Koka A., Hein V., Tilga H., Raudsepp L. (2020). Motivational Processes in Physical Education and Objectively Measured Physical Activity among Adolescents. J. Sport Health Sci..

[7] Vasconcellos D., Parker P.D., Hilland T., Cinelli R., Owen K., Kapsal N., Lonsdale C. (2020). Self-Determination Theory Applied to Physical Education: A Systematic Review and Meta-Analysis. J. Educ. Psychol..

[8] Ryan R., Deci E.L. (2000). La Teoría de La Autodeterminación y La Facilitación de La Motivación Intrínseca, El Desarrollo Social, y El Bienestar. Am. Psychol..

[9] Markland D., Tobin V.J. (2010). Need Support and Behavioral Regulations for Exercise among Exercise Referral Scheme Clients: The Mediating Role of Psychological Need Satisfaction. Psychol. Sport Exerc..

[10] Ng J.Y.Y., Ntoumanis N., Thøgersen-Ntoumani C., Deci E.L., Ryan R.M., Duda J.L., Williams G.C. (2012). Self-Determination Theory Applied to Health Contexts: A Meta-Analysis. Perspect. Psychol. Sci..

[11] Ryan R.M., Connell J.P. (1989). Perceived Locus of Causality and Internalization: Examining Reasons for Acting in Two Domains. J. Pers. Soc. Psychol..

[12] Lonsdale C., Hodge K., Rose E.A. (2008). The Behavioral Regulation in Sport Questionnaire (BRSQ): Instrument Development and Initial Validity Evidence. J. Sport Exerc. Psychol..

[13] Mullan E., Markland D., Ingledew D.K. (1997). A Graded Conceptualization of Self-Determination in the Regulation of Exercise Behavior: Development of a Measure Using Confirmatory Factor Analytic Procedures. Personal. Individ. Differ..

[14] Markland D., Tobin V. (2004). A Modification to the Behavioral Regulation in Exercise Questionnaire to Include an Assessment of Amotivation. J. Sport Exerc. Psychol..

[15] González-Cutre D., Sicilia A., Fernández A. (2010). Hacia Una Mayor Comprensión de La Motivación En El Ejercicio Físico: Medición de La Regulación Integrada En El Contexto Español. Psicothema.

[16] Reyes-Molina D., Nazar G., Cigarroa I., Carrasco-Marín F., Cárcamo-Regla R., Rozas Pardo K., Zapata-Lamana R. (2023). Motivación, Barreras y Beneficios Para La Práctica de Ejercicio Físico En Una Intervención Mobile Health En Adultos Del Biobío, Chile. Retos Nuevas Perspect. Educ. Física Deporte Recreación.

[17] Mella Norambuena J.A., Nazar Carter G., Sáez Delgado F., Bustos Navarrete C., López-Angulo Y., Cobo Rendón R. (2020). Variables Sociocognitivas y Su Relación Con La Actividad Física En Estudiantes Universitarios Chilenos (Sociocognitive Variables and Their Relationship with Physical Activity in Chilean University Students). Retos.

[18] Cid L., Monteiro D., Teixeira D., Teques P., Alves S., Moutão J., Silva M., Palmeira A. (2018). The Behavioral Regulation in Exercise Questionnaire (BREQ-3) Portuguese-Version: Evidence of Reliability, Validity and Invariance Across Gender. Front. Psychol..

[19] Cavicchiolo E., Sibilio M., Lucidi F., Cozzolino M., Chirico A., Girelli L., Manganelli S., Giancamilli F., Galli F., Diotaiuti P. (2022). The Psychometric Properties of the Behavioral Regulation in Exercise Questionnaire (BREQ-3): Factorial Structure, Invariance and Validity in the Italian Context. Int. J. Environ. Res. Public Health.

[20] Guedes D., Sofiati S. (2015). Tradução e Validação Psicométrica Do Behavioral Regulation in Exercise Questionnaire Para Uso Em Adultos Brasileiros. Rev. Bras. Atividade Física Saúde.

[21] Palombi T., Lucidi F., Chirico A., Alessandri G., Filosa L., Tavolucci S., Borghi A.M., Fini C., Cavicchiolo E., Pistella J. (2023). Is the Behavioral Regulation in Exercise Questionnaire a Valid Measure in Older People?. Healthcare.

[22] De Oliveira Vanini J., Karloh M., Coelho Bosco R., De Souza M.G., Karsten M., Matte D.L. (2025). The Brazilian Portuguese Version of the Behavioral Regulation in Exercise Questionnaire 3 (BREQ-3) Is Reliable and Valid for Assessing Motivational Regulations and Self-Determination in Exercise Among Adults Aged 50 Years or Older: A Methodological Study. Int. J. Environ. Res. Public Health.

[23] Comeau E. (2021). Motivation and Behavioral Regulations of Children and Youth Related to Physical Activity Intensity During the COVID-19 Pandemic. Master’s Thesis.

[24] Serrano Valenzuela J.L., Villegas Toro M.D.L.Á., Gallardo Navarro I. (2023). El Ejercicio Físico y Regulación de La Conducta En Niños y Niñas de 10 a 13 Años Del Sector Rural y Urbano de La Comuna de Cartagena, Chile. Rev. Obs. Deporte.

[25] Lansford J.E., French D.C., Gauvain M. (2021). Child and Adolescent Development in Cultural Context.

[26] Shandilya S., Devanesan P. (2020). Culture and Adolescent’s Socio Cognitive Development. SSRN Electron. J..

[27] Cadime I., Ribeiro I., Lorusso M.L. (2024). Cognitive and Linguistic Development in Children and Adolescents. Children.

[28] McRae K., Gross J.J., Weber J., Robertson E.R., Sokol-Hessner P., Ray R.D., Gabrieli J.D.E., Ochsner K.N. (2012). The Development of Emotion Regulation: An fMRI Study of Cognitive Reappraisal in Children, Adolescents and Young Adults. Soc. Cogn. Affect. Neurosci..

[29] Green S.B., Akey T.M., Fleming K.K., Hershberger S.L., Marquis J.G. (1997). Effect of the Number of Scale Points on Chi-square Fit Indices in Confirmatory Factor Analysis. Struct. Equ. Model. A Multidiscip. J..

[30] Xia Y., Yang Y. (2019). RMSEA, CFI, and TLI in Structural Equation Modeling with Ordered Categorical Data: The Story They Tell Depends on the Estimation Methods. Behav. Res. Methods.

[31] Wells C.S. (2021). Assessing Measurement Invariance for Applied Research.

[32] Glen S. Elementary Statistics for the Rest of Us! Cronbach’s Alpha: Definition, Interpretation, SPSS. www.statisticshowto.com/probability-and-statistics/statistics-definitions/cronbachs-alpha-spss.

[33] Shrout P.E., Fleiss J.L. (1979). Intraclass Correlations: Uses in Assessing Rater Reliability. Psychol. Bull..

[34] Weir J.P. (2005). Quantifying Test-Retest Reliability Using the Intraclass Correlation Coefficient and the SEM. J. Strength. Cond. Res..

[35] Kline R.B. (2023). Principles and Practice of Structural Equation Modeling.

[36] Hu L.T., Bentler P.M. (1999). Cutoff Criteria for Fit Indices in Covariance Structure Analysis: Conventional Criteria versus New Alternatives. Struct. Equ. Model. Multidiscip. J..

[37] Mw B. (1993). Alternative Ways of Assessing Model Fit. Testing Structural Equation Models.

[38] Chai S., Kueh Y.C., Majdi Yaacob N., Kuan G. (2022). Psychometric Properties of the Malay Version of the Behavioral Regulation in Exercise Questionnaire (BREQ-3). PLoS ONE.

[39] Cocca A., Kopp M., Greier K., Labek K., Cocca M., Ruedl G. (2024). Validity, Reliability, and Invariance across Sex of a German Version of the Behavioral Regulation in Exercise Questionnaire. Front. Psychol..

[40] Eleutério S., Rebustini F. (2024). Evidências De Validade De Estrutura Interna Do Behavioral Regulation In Exercise Questionnaire (Brsq-3) Para A População Idosa Brasileira. Rev. Interfaces Saúde Humanas Tecnol..

[41] Marsh H.W., Shavelson R. (1985). Self-Concept: Its Multifaceted, Hierarchical Structure. Educ. Psychol..

[42] Standage M., Duda J.L., Ntoumanis N. (2006). Students’ Motivational Processes and Their Relationship to Teacher Ratings in School Physical Education: A Self-Determination Theory Approach. Res. Q. Exerc. Sport.

[43] Hattie J., Hodis F.A., Kang S.H.K. (2020). Theories of Motivation: Integration and Ways Forward. Contemp. Educ. Psychol..

[44] Barkoukis V., Tsorbatzoudis H., Grouios G. (2008). Manipulation of Motivational Climate in Physical Education: Effects of a Seven-Month Intervention. Eur. Phys. Educ. Rev..

[45] Dunton G.F., Huh J., Leventhal A.M., Riggs N., Hedeker D., Spruijt-Metz D., Pentz M.A. (2014). Momentary Assessment of Affect, Physical Feeling States, and Physical Activity in Children. Health Psychol..

[46] Pastor-Cisneros R., Mendoza-Muñoz M., Carlos-Vivas J., Adsuar-Sala J.C., López-Gil J.F., SPLA-C Expert Committee (2025). Developing the Spanish Physical Literacy Assessment for Children (SPLA-C): A Delphi-Based Model for Assessing Physical Literacy in Schoolchildren Aged 6–12 Years. Eur. J. Pediatr..

