# Supplementary material for: Cross-Cultural Adaptation and Validation of the Spanish Version of the Behavioral Regulation in Exercise Questionnaire for Children (BREQ-3C): Analysis of Psychometric Properties
Source: Healthcare (Basel). 2025 Sep 2;13(17):2197. doi: 10.3390/healthcare13172197 (PMC12427641; doi:10.3390/healthcare13172197)
Supplement: Supplementary file 1 [file healthcare-13-02197-s001.zip › healthcare-3768780-supplementary.pdf]

CUESTIONARIO DE REGULACIÓN DE LA CONDUCTA EN EL EJERCICIO FÍSICO PARA NIÑOS (BREQ-3C).

**Código:**	*Nada verdadero*				*Totalmente verdadero*
1. Yo hago ejercicio/deporte porque los/las demás me dicen que tengo que hacerlo	0	1	2	3	4
2. Yo hago ejercicio/deporte porque me siento mal cuando no lo hago	0	1	2	3	4
3. Yo hago ejercicio/deporte porque sé que es bueno para mí	0	1	2	3	4
4. Yo hago ejercicio/deporte porque creo que es divertido	0	1	2	3	4
5. Yo hago ejercicio/deporte porque va bien con mi forma de ser	0	1	2	3	4
6. No creo que tenga que hacer deporte/ejercicio	0	1	2	3	4
7. Yo hago ejercicio/deporte porque mis amigos y mi familia me dicen que tengo que hacerlo	0	1	2	3	4
8. Yo hago ejercicio/deporte porque me da vergüenza si no voy a un entrenamiento/clase	0	1	2	3	4
9. Yo hago ejercicio/deporte porque para mí es importante hacerlo cada semana	0	1	2	3	4
10. Yo hago ejercicio/deporte porque creo que es parte de mi	0	1	2	3	4
11. No entiendo por qué tengo que hacer ejercicio o deporte	0	1	2	3	4
12. Yo hago ejercicio/deporte porque disfruto haciéndolo	0	1	2	3	4

13. Yo hago ejercicio/deporte porque a los/las demás no les gustará si no lo hago	0	1	2	3	4
14. No veo por qué es importante hacer ejercicio	0	1	2	3	4
15. Yo hago ejercicio/deporte porque veo que es una parte muy importante de mí	0	1	2	3	4
16. Yo hago ejercicio/deporte porque me siento mal si no lo hago	0	1	2	3	4
17. Yo hago ejercicio/deporte porque creo que es importante esforzarse para hacerlo cada semana	0	1	2	3	4
18. Yo hago ejercicio/deporte porque me gusta mucho hacerlo	0	1	2	3	4
19. Yo hago ejercicio/deporte porque mis amigos o familia me obligan a hacerlo	0	1	2	3	4
20. Yo hago ejercicio/deporte porque creo que va con lo que pienso que es correcto	0	1	2	3	4
21. Yo hago ejercicio/deporte porque me pongo nervioso/a si no lo hago cada semana	0	1	2	3	4
22. Yo hago ejercicio/deporte porque me hace sentir bien	0	1	2	3	4
23. Pienso que hacer ejercicio deporte no vale la pena	0	1	2	3	4

BEHAVIORAL REGULATION IN EXERCISE QUESTIONNAIRE FOR CHILDREN (BREQ-3C).

English version. Please note that this version has not been adapted or validated.

Code:	Nothing true				Totally true
1. I do exercise/sport because others tell me I have to do it	0	1	2	3	4
2. I do exercise/sport because I feel bad when I don't do it.	0	1	2	3	4
3. I do exercise/sport because I know it is good for me.	0	1	2	3	4
4. I do exercise/sport because I think it's fun.	0	1	2	3	4
5. I do exercise/sport because it suits my personality.	0	1	2	3	4
6. I don't think I have to do sport/exercise.	0	1	2	3	4
7. I do exercise/sport because my friends and family tell me I have to.	0	1	2	3	4
8. I do exercise/sport because I am embarrassed if I don't go to a training/class.	0	1	2	3	4
9. I do exercise/sport because it is important to me to do it every week.	0	1	2	3	4
10. I do exercise/sport because I believe it is part of me.	0	1	2	3	4
11. I don't understand why I have to do exercise or sport.	0	1	2	3	4
12. I do exercise/sport because I enjoy doing it.	0	1	2	3	4

13. I do exercise/sport because others won't like it if I don't do it.	0	1	2	3	4
14. I don't see why it is important to exercise.	0	1	2	3	4
15. I do exercise/sport because I see it as a very important part of me.	0	1	2	3	4
16. I do exercise/sport because I feel bad if I don't do it.	0	1	2	3	4
17. I do exercise/sport because I believe it is important to make the effort to do it every week.	0	1	2	3	4
18. I do exercise/sport because I like to do it a lot.	0	1	2	3	4
19. I do exercise/sport because my friends or family make me do it.	0	1	2	3	4
20. I do exercise/sport because I think it goes with what I think is right.	0	1	2	3	4
21. I do exercise/sport because I get nervous if I don't do it every week.	0	1	2	3	4
22. I do exercise/sport because it makes me feel good.	0	1	2	3	4
23. I think that doing sport is not worth it.	0	1	2	3	4
